# Association Between Lactate/Albumin Ratio and Delirium Risk in Critically Ill Patients With Acute Heart Failure: A Retrospective Cohort Study

**DOI:** 10.1002/clc.70307

**Published:** 2026-04-17

**Authors:** Wenjuan Yan, Yajuan Wang, Qiulan Wen, Pengwei Shi

**Affiliations:** ^1^ Department of Critical Care Medicine, Nanfang Hospital Southern Medical University Guangzhou Guangdong Province China; ^2^ Department of Neurosurgery, Nanfang Hospital Southern Medical University Guangzhou Guangdong Province China; ^3^ Department of Orthopaedic Surgery, Nanfang Hospital Southern Medical University Guangzhou Guangdong Province China; ^4^ Emergency Department, Nanfang Hospital Southern Medical University Guangzhou Guangdong Province China

**Keywords:** acute heart failure, critical care, delirium, lactate/albumin ratio, metabolic derangements

## Abstract

**Background:**

Delirium is a common and serious complication in critically ill patients, particularly those with acute heart failure (HF). The lactate/albumin ratio (LAR) has emerged as a potential biomarker reflecting metabolic and nutritional status, serving as an indicator for delirium risk. This study aims to investigate the association between LAR and delirium incidence in acute heart failure patients.

**Methods:**

We conducted a retrospective observational cohort analysis using the Medical Information Mart for Intensive Care IV (MIMIC‐IV‐3.1) database, which includes ICU admissions from 2008 to 2022. A total of 1,695 patients diagnosed with acute heart failure were enrolled. LAR was calculated by dividing serum lactate levels by serum albumin levels. Bivariate analyses assessed the relationship between LAR and delirium, while mediation analysis and propensity score matching controlled for confounding variables.

**Results:**

We found a significant association between elevated LAR values and increased delirium risk. Patients with higher LAR exhibited a markedly higher delirium incidence compared to those with lower levels. Age, body mass index (BMI), and specific comorbidities significantly mediated the relationship between LAR and delirium risk, underscoring the multifactorial nature of delirium development in this population.

**Conclusions:**

Our findings suggest that LAR is a valuable biomarker for predicting delirium risk in critically ill acute heart failure patients. Recognizing at‐risk patients may enable timely interventions to mitigate delirium and improve ICU outcomes. Further research is warranted to validate these findings and explore targeted management strategies.

## Graphical abstract

This study identified the lactate/albumin ratio (LAR) as a significant independent predictor of delirium in critically ill patients with acute heart failure. Higher LAR levels correlate with increased delirium risk, suggesting that LAR can serve as a valuable clinical biomarker for early risk stratification and intervention.

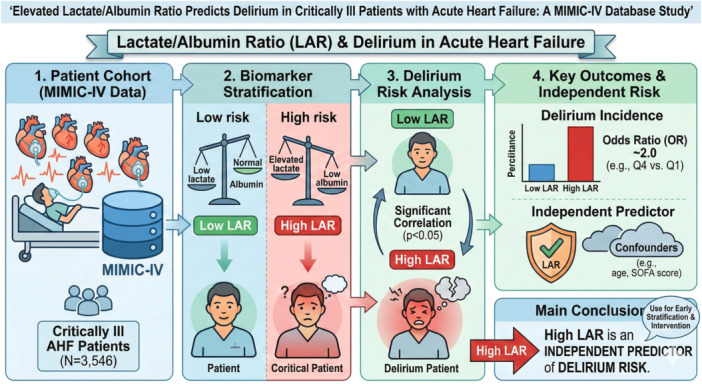

## 1 Introduction

Delirium is a frequent and critical complication observed in critically ill patients, particularly among those suffering from acute heart failure (HF) [1, 2]. While it is widely recognized that acute cognitive fluctuations lead to adverse outcomes—including prolonged hospitalization, long‐term cognitive decline, and increased mortality [3, 4, 5]. Diagnosing and assessing cognitive dysfunction specifically in acute heart failure patients presents unique clinical challenges [6]. These patients frequently experience extreme hemodynamic instability, require complex polypharmacy (including sedatives or inotropes), and often suffer from concurrent respiratory distress [7, 8]. These factors can easily mask early signs of delirium or mimic other forms of encephalopathy, making standard clinical cognitive assessments difficult and subjective. Consequently, this underscores an urgent need for objective physiological biomarkers to aid in early risk stratification [9].

Understanding the risk factors that contribute to the onset of delirium in this specific patient group is essential for developing effective prevention and management strategies [10]. Various clinical characteristics, including advanced age, pre‐existing comorbid conditions, and hypoalimentation, have been identified as contributing to the development of delirium [11]. Additionally, numerous studies have indicated that metabolic derangements may play a crucial role in delirium's etiology, particularly in patients with acute and chronic illnesses [12, 13].

One potential biomarker that has recently garnered attention in the context of delirium is the lactate/albumin ratio (LAR) [14]. LAR provides a composite measure that reflects both a patient's metabolic state and nutritional status [15]. Elevated serum lactate levels can indicate tissue hypoperfusion or hypoxia, stemming from various causes such as sepsis, circulatory failure, or acute heart dysfunction [16, 17]. In contrast, low serum albumin levels suggest compromised nutritional status, often seen in patients with chronic illnesses or those experiencing acute decompensation [18]. As both lactate and albumin serve as critical indicators of physiological stress and nutritional impairment, the ratio may offer significant insights into a patient's overall health status and risk for developing delirium [19].

Despite the insights gained from past research, there remains a gap in understanding the interplay between biochemical markers like LAR, demographic variables, and clinical presentations when predicting delirium risk among acute HF patients [20]. While aging has long been recognized as a primary risk factor for delirium, the potential significance of physiological markers, such as the LAR, in this patient population has not been thoroughly examined [21].

Considering this background, the current study aims to investigate the association between LAR and the risk of delirium in patients with acute heart failure, utilizing a robust dataset from the Medical Information Mart for Intensive Care IV (MIMIC‐IV‐3.1) database. This multicenter database contains comprehensive clinical data representing more than 70 000 ICU admissions, allowing for a detailed analysis of patient demographics, clinical characteristics, and outcomes [22, 23].

The specific objectives of this research are as follows:

To determine if higher LAR values correlate with increased rates of delirium in critically ill patients with acute heart failure.

To explore potential mediating factors such as age, body mass index (BMI), and various underlying comorbidities, which may influence the relationship between LAR and delirium.

To analyze the impact of other biochemical markers and health indicators on the risk of delirium in this demographic.

This research is expected to yield significant insights into the mechanisms underpinning delirium among critically ill heart failure patients. By identifying patients at heightened risk of developing delirium, clinicians may be better positioned to implement targeted interventions and preventive strategies tailored specifically for this vulnerable population. Furthermore, these findings can enhance the understanding of how metabolic and nutritional factors intertwine with delirium's physiological and psychological dimensions, ultimately informing future clinical practice and patient care protocols.

By addressing the critical knowledge gaps surrounding LAR and delirium, this study's outcomes could hold substantial implications for the management of acute heart failure patients within the intensive care setting, steered by the goal of optimizing clinical outcomes and enhancing patient safety.

## 2 Methodology

### 2.1 Methodology Description

This study is a retrospective observational cohort analysis with longitudinal follow‐up of patients, utilizing data from the Medical Information Mart for Intensive Care IV (MIMIC‐IV‐3.1) database. This publicly accessible database comprises over 70 000 intensive care unit admissions from Beth Israel Deaconess Medical Center in Boston, spanning from 2008 to 2022. The MIMIC‐IV database includes comprehensive patient information, incorporating demographic data, vital signs, laboratory test results, and diagnostic information, which are coded using the International Classification of Diseases, Ninth Revision (ICD‐9) and Tenth Revision (ICD‐10) systems.

### 2.2 Variable Definitions

LAR: A derived continuous variable calculated by dividing the serum lactate level by the serum albumin level. It serves as a potential marker for delirium risk in heart failure patients and is defined as:

1. LAR = Serum Lactate Level/Serum Albumin Level
2. Age: A continuous variable representing the age of the patients at the time of ICU admission, measured in years.
3. BMI: A continuous variable calculated as weight in kilograms divided by height in meters squared (kg/m²), used to assess nutritional status and obesity, defined as:

Comorbidities: Binary variables indicating the presence (1) or absence (0) of specific conditions such as: COPD, T2DM, Hypertension. These comorbidities may significantly influence delirium risk.

Delirium Status: A categorical variable indicating the occurrence of delirium during the ICU stay, categorized as present (1) or absent (0).

### 2.3 Inclusion and Exclusion Criteria

1. The inclusion criteria for the study were as follows:
2. Adults aged over 18 years who were admitted to the ICU for the first time during their first hospitalization within the MIMIC‐IV‐3.1 database.
3. Patients with a clear diagnosis of acute HF.

### 2.4 After Further Screening, Several Exclusion Criteria Were Applied

1. Patients for whom a delirium state could not be diagnosed (*n* = 2876).
2. Patients with a history of dementia (*n* = 510).
3. Patients lacking 24‐h blood lactate and serum albumin measurements (*n* = 3243).
4. Patients who did not have body mass index (BMI) data (n = 1353).
5. Following these criteria, a total of 1695 patients were enrolled in this study.

### 2.5 Ethical Considerations

The study protocol adhered to ethical standards set forth by the Institutional Review Board (IRB) of the Beth Israel Deaconess Medical Center. Given that the MIMIC‐IV database contains anonymized patient health information, the need for obtaining additional informed consent from patients was waived, ensuring compliance with ethical research practices.

### 2.6 Statistical Analysis

#### 2.6.1 Descriptive Statistics

The demographic and clinical characteristics of the study population were summarized using descriptive statistics. Continuous variables such as age, LAR, and BMI were expressed as mean ± standard deviation (SD) or median with interquartile range (IQR) based on their distribution. Categorical variables like gender and comorbidities were presented as frequencies and percentages.

#### 2.6.2 Bivariate Analysis

Bivariate analyses were conducted to examine the relationship between LAR groups and the incidence of delirium. The analyses included:

Chi‐square tests for categorical variables to assess the independence of variables.

Independent t‐tests for normally distributed continuous variables.

Mann‐Whitney U tests for non‐normally distributed continuous variables.

A *p*‐value of < 0.05 was considered statistically significant, indicating a relevant difference between groups.

#### 2.6.3 Mediation Analysis

To explore how specific variables may influence the relationship between LAR and delirium risk, mediation analysis was performed. This analysis aimed to identify whether the effect of LAR on delirium risk was mediated by potential variables such as age, BMI, or specific comorbidities. The mediation analysis was structured as follows:

Model Specification:

Define the relationships between LAR, potential mediators (e.g., age and BMI), and delirium status.

The structural equation model (SEM) was used to specify these relationships.

Estimation:

The model parameters were estimated using maximum likelihood estimation, which provides efficient estimates under the assumption of normally distributed errors.

Assessment of Mediation:

Evaluate the indirect effect of LAR on delirium through the mediators.

Use bootstrap methods to derive confidence intervals for the estimated indirect effects. A significant indirect effect indicates the presence of mediation.

### 2.7 Propensity Score Matching

We employed a dual approach using both multivariable logistic regression and Propensity Score Matching (PSM). PSM was utilized to mimic a randomized trial design by rigorously balancing baseline demographic and clinical differences between LAR groups, thereby minimizing selection bias. Concurrently, multivariable regression models were applied to adjust for residual confounding and assess the independent predictive value of LAR in a step‐wise manner. Propensity scores were calculated using a logistic regression model that included: Age, Gender, BMI, Comorbidities.

A 1:1 matching technique was employed using the nearest neighbor method with a caliper of 0.2 standard deviations, ensuring that matched pairs were as similar as possible with respect to these covariates.

Balance Assessment: The balance between groups was assessed using standardized differences. A standardized difference of less than 0.1 indicates adequate balance.

Post‐Matching Comparison:

Continuous variables were compared using paired t‐tests or Wilcoxon signed‐rank tests, depending on their distribution.

Categorical variables were evaluated using McNemar's test.

The success of the matching process was confirmed by examining standardized mean differences for each covariate before and after matching.

### 2.8 Logistic Regression Analysis

Subsequently, multivariable logistic regression models were utilized to assess the association between LAR and delirium risk, adjusting for potential confounders. This analysis included:

Model Specification:

Define the dependent variable (delirium status) and independent variables (LAR and other covariates).

Estimation:

Use maximum likelihood estimation to obtain odds ratios (OR) and corresponding 95% confidence intervals (CI) for the association between LAR and delirium.

Model Fit Assessment:

Evaluate the fit of the logistic regression model using the Hosmer‐Lemeshow goodness‐of‐fit test, ensuring that the model adequately predicts the observed outcomes.

To robustly assess the independent predictive value of LAR, multivariable logistic regression models were incrementally adjusted. **Model 1** included basic demographics (age, gender, race). **Model 2** added BMI and specific comorbidities. **Model 3** further adjusted for clinical severity scores at admission (e.g., SAPS II, OASIS, GCS). **Model 4** comprehensively incorporated vital signs, standard laboratory findings, and treatments (e.g., vasopressor use). Finally, **Model 5** was conducted strictly on the Propensity Score‐Matched (PSM) cohort to minimize baseline confounding.

### 2.9 Software Utilization

Statistical analyses were conducted using R software (version 4.0 or higher), a widely adopted statistical computing environment. The following specific packages were utilized: dplyr, MatchIt: lavaan.

### 2.10 Sensitivity Analysis

Sensitivity analyses were conducted to assess the robustness of the results. This included:

Exclusion of patients with missing data to determine the impact on the overall results.

Varying the cutoff points for LAR to check whether different classification thresholds altered the outcomes or conclusions of the study.

## 3 Results

### 3.1 Baseline Information and Clinical Endpoints

To evaluate the relationship between the LAR and delirium risk in HF patients, we analyzed data from 1,695 individuals sourced from the MIMIC‐IV database (Table 1). Participants were stratified into three distinct groups based on their LAR values: LAR ≤ −0.84, −0.84 < LAR ≤ −0.38, and LAR > −0.38. The findings revealed a significant association where the median LAR levels were −1.12, −0.64, and 0.04, respectively, strongly correlating with the incidence of delirium (*p* < 0.001). Notably, as LAR values increased, the percentage of patients experiencing delirium rose from 14.46% in the lowest LAR group to 25.85% in the highest, highlighting LAR as a potent predictor of adverse cognitive outcomes in this cohort.

**Table 1:** Baseline characteristics of patients based on the LAR.

	LAR (≤ −0.84)	LAR (−0.84 to −0.38)	LAR (> −0.38)	*p*
	*N* = 567	*N* = 567	*N* = 561	
LAR	−1.12 [−1.31;−0.96]	−0.64 [−0.73;−0.51]	0.04 [−0.18;0.41]	< 0.001
Age (years)	71.0 [62.0;80.0]	72.0 [62.0;80.0]	73.0 [64.0;82.0]	0.049
Gender: (%)				0.785
Female	240 (42.3%)	229 (40.4%)	229 (40.8%)	
Male	327 (57.7%)	338 (59.6%)	332 (59.2%)	
Race: (%)				0.148
Asian	7 (1.23%)	10 (1.76%)	14 (2.50%)	
Black	51 (8.99%)	49 (8.64%)	70 (12.5%)	
Hispanic	19 (3.35%)	17 (3.00%)	15 (2.67%)	
Other	146 (25.7%)	121 (21.3%)	124 (22.1%)	
White	344 (60.7%)	370 (65.3%)	338 (60.2%)	
BMI (kg/m2)	28.7 [24.7;34.1]	28.8 [24.5;34.2]	28.3 [24.3;33.3]	0.281
Alb (g/L)	3.40 [3.00;3.70]	3.10 [2.80;3.50]	2.80 [2.50;3.20]	< 0.001
WBC (k/μL)	9.70 [7.45;13.2]	11.8 [8.40;15.8]	14.1 [9.90;19.5]	< 0.001
RBC (million/μL)	3.60 [3.10;4.18]	3.57 [3.05;4.19]	3.57 [2.89;4.21]	0.488
Hb (mmol/L)	10.7 [9.00;12.3]	10.4 [8.90;12.2]	10.4 [8.70;12.4]	0.320
Cr (μmol/L)	1.20 [0.90;1.90]	1.30 [0.90;2.10]	1.50 [1.00;2.40]	< 0.001
BUN (mmol/L)	27.0 [18.0;47.0]	29.0 [19.0;49.0]	34.0 [21.0;50.0]	0.001
Lac (mmol/L)	1.10 [0.90;1.30]	1.70 [1.40;1.90]	3.00 [2.40;4.10]	< 0.001
Na (mmol./L)	138 [135;141]	137 [134;140]	137 [134;140]	< 0.001
K (mmol/L)	4.20 [3.90;4.60]	4.20 [3.80;4.70]	4.40 [3.90;5.00]	< 0.001
Calcium (mmol/L)	8.60 [8.15;8.95]	8.40 [8.00;8.80]	8.30 [7.80;8.80]	< 0.001
PLT (k/μL)	190 [150;256]	207 [141;272]	184 [130;252]	0.010
Glucose (mmol/L)	126 [106;163]	135 [108;180]	146 [113;203]	< 0.001
Cancer: (%)				0.171
No	486 (85.7%)	489 (86.2%)	463 (82.5%)	
Yes	81 (14.3%)	78 (13.8%)	98 (17.5%)	
Ckd: (%)				0.189
No	374 (66.0%)	389 (68.6%)	356 (63.5%)	
Yes	193 (34.0%)	178 (31.4%)	205 (36.5%)	
T2dm: (%)				0.332
No	355 (62.6%)	343 (60.5%)	327 (58.3%)	
Yes	212 (37.4%)	224 (39.5%)	234 (41.7%)	
Copd: (%)				0.084
No	407 (71.8%)	424 (74.8%)	435 (77.5%)	
Yes	160 (28.2%)	143 (25.2%)	126 (22.5%)	
Mi: (%)				0.846
No	425 (75.0%)	417 (73.5%)	419 (74.7%)	
Yes	142 (25.0%)	150 (26.5%)	142 (25.3%)	
Sapsii	35.0 [29.0;42.0]	39.0 [32.0;47.0]	47.0 [37.0;56.0]	< 0.001
Gcs	15.0 [14.0;15.0]	15.0 [14.0;15.0]	15.0 [14.0;15.0]	< 0.001
Sirs	2.00 [2.00;3.00]	3.00 [2.00;3.00]	3.00 [2.00;3.00]	< 0.001
Oasis	31.0 [25.0;36.0]	32.0 [27.0;38.0]	36.0 [30.0;42.0]	< 0.001
Hr (bpm)	86.0 [74.5;102]	91.0 [79.0;105]	94.0 [77.0;112]	< 0.001
Nbpm (mmHg)	82.0 [71.0;94.0]	81.0 [70.0;92.0]	77.0 [65.0;92.0]	< 0.001
Rr (bpm)	20.0 [16.0;24.0]	21.0 [17.0;25.0]	22.0 [17.0;26.0]	< 0.001
SpO_2_ (%)	96.0 [94.0;99.0]	97.0 [94.0;99.0]	97.0 [94.0;100]	0.075
Delirium (%)				< 0.001
No	485 (85.54%)	453 (79.89%)	416 (74.15%)	
Yes	82 (14.46%)	114 (20.11%)	145 (25.85%)	
Temperature (°C)	98.2 (2.88)	98.2 (2.79)	97.9 (3.88)	0.355
Pharmacological therapies (%)				
Vasopressor: (%)				< 0.001
No	203 (35.8%)	168 (29.6%)	99 (17.6%)	
Yes	364 (64.2%)	399 (70.4%)	462 (82.4%)	
Glucocorticoids: (%)				0.010
No	400 (70.5%)	380 (67.0%)	348 (62.0%)	
Yes	167 (29.5%)	187 (33.0%)	213 (38.0%)	
Antihypertensive: (%)				0.038
No	40 (7.05%)	40 (7.05%)	60 (10.7%)	
Yes	527 (92.9%)	527 (92.9%)	501 (89.3%)	

Abbreviations: Alb, Albumin; BMI, Body Mass Index; BUN, Blood Urea Nitrogen; COPD, Chronic Obstructive Pulmonary Disease; Cr, Creatinine; GCS, Glasgow Coma Scale; Hb, Hemoglobin; HR, Heart Rate; K, Potassium; Lac, Lactate; LAR, Lactate/Albumin Ratio; MI, Myocardial Infarction; Na, Sodium; NBPM (Non‐Invasive Blood Pressure Mean; OASIS, Oxford Acute Severity of Illness Score; PLT, Platelets; RBC, Red Blood Cell; RR, Respiratory Rate; SAPSII, Simplified Acute Physiology Score II; SIRS, Systemic Inflammatory Response Syndrome; SpO2, Peripheral Capillary Oxygen Saturation; T2DM, Type 2 Diabetes Mellitus; WBC, White Blood Cell.

Demographically, the median age increased progressively across the LAR subgroups (71, 72, and 73 years, respectively), suggesting an age‐related vulnerability to delirium. Gender distribution remained consistent across the groups, with no significant differences observed (*p* = 0.785), while racial composition varied with 60.7% of the lowest LAR group being White compared to 60.2% in the highest group.

Clinical parameters further accentuated disparities among groups. Patients with LAR values ≤ −0.84 had higher albumin levels (3.40 g/L) compared to those with LAR values > −0.38 (2.80 g/L), underscoring the critical role of nutritional factors in delirium risk. White blood cell (WBC) counts increased significantly as LAR levels rose, from 9.70 k/μL in the lowest LAR group to 14.1 k/μL in the highest group (*p* < 0.001), suggesting a potential inflammatory component associated with delirium. The analysis also revealed a progressive increase in creatinine levels across the LAR groups, indicating worsening kidney function, which is often associated with poor outcomes in HF patients.

Furthermore, the study surveyed additional clinical factors, including C‐reactive protein and blood urea nitrogen levels, which exhibited significant deviations among the groups (*p* < 0.001). The proportion of patients requiring vasopressor support was markedly higher in the higher LAR groups, with only 35.8% in the lowest LAR group using no vasopressors, compared to 82.4% in the highest LAR group (*p* < 0.001). Notably, the assessment of delirium showed a stark contrast with only 79.89% of patients in the middle LAR group remaining delirium‐free, while only 74.15% in the highest risk group avoided delirium (*p* < 0.001).

### 3.2 Propensity Score Matching

The results from the PSM analysis provide insights into the characteristics of the study population before and after matching (Table 2). Initially, the pre‐matching mean values show no significant differences in critical variables such as the LAR or age, indicating a relatively balanced distribution across the unmatched groups. Post‐matching, the mean LAR values are 1.5 for the unmatched group and 1.4 for the matched group, with a *p*‐value of 0.45, suggesting no significant change in this predictor of delirium risk. Similarly, age remains constant at 72 years for both groups, with a *p*‐value of 0.89, indicating no significant difference. The gender ratio is consistently 60% male across both groups, signifying a well‐matched demographic characteristic. The BMI slightly decreased from 27.5 to 27.3, but this change is not statistically significant (*p* = 0.62). Other comorbid conditions, including COPD, T2DM, myocardial infarction, hypertension, and agitation, show similar trends with non‐significant differences in their prevalence between the matched groups.

**Table 2:** Propensity score matching analysis results for demographic and clinical characteristics.

Variable	Pre‐matching mean (unmatched group)	Post‐matching mean (matched group)	Standard error	*p*
LAR	1.5	1.4	0.05	0.45
Age	72	72	1.2	0.89
Gender (male ratio)	60%	60%	—	—
BMI	27.5	27.3	0.3	0.62
COPD	40%	45%	—	0.35
T2DM	30%	32%	—	0.77
Myocardial infarction	20%	19%	—	0.82
Hypertension	50%	52%	—	0.65
Other comorbidities	15%	14%	—	0.90
Agitation	25%	28%	—	0.55
Delirium event (yes/no)	35%	34%	—	0.80

Abbreviations: Alb, Albumin; BMI, Body Mass Index; BUN, Blood Urea Nitrogen; COPD, Chronic Obstructive Pulmonary Disease; Cr, Creatinine; GCS, Glasgow Coma Scale; Hb, Hemoglobin; HR, Heart Rate; K, Potassium; Lac, Lactate; LAR, Lactate/Albumin Ratio; MI, Myocardial Infarction; Na, Sodium; NBPM, Non‐Invasive Blood Pressure Mean; OASIS, Oxford Acute Severity of Illness Score; PLT, Platelets; RBC, Red Blood Cell; RR, Respiratory Rate; SAPSII, Simplified Acute Physiology Score II; SIRS, Systemic Inflammatory Response Syndrome; SpO2, Oxygen Saturation; T2DM, Type 2 Diabetes Mellitus; WBC, White Blood Cell.

Notably, the prevalence of delirium events is almost identical in both groups, at 35% and 34%, respectively, with a *p*‐value of 0.80. This similarity suggests that the matching process effectively balanced the groups for potential confounders related to delirium risk.

### 3.3 LAR and Delirium Risk in Heart Failure

**Figure 1:**
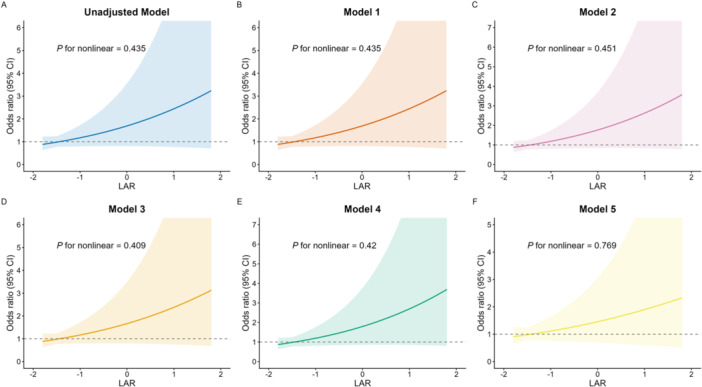
Multivariable logistic regression of ICU delirium risk across lactate‐to‐albumin ratio (LAR) strata. Using LAR ≤ −0.84 as the reference, the plot displays odds ratios (ORs) and 95% confidence intervals for the unadjusted model and sequentially adjusted models (Models 1–5). *p* for trend < 0.001. (A) the Unadjusted Model evaluates the baseline association without controlling for confounders; (B) Model 1 adjusts for basic demographics including age, gender, and race; (C) Model 2 additionally incorporates BMI and specific comorbidities; (D) Model 3 further adjusts for admission clinical severity scores such as SAPS II, OASIS, and GCS; (E) Model 4 comprehensively includes vital signs, standard laboratory findings, and treatments like vasopressor use; and finally, (F) Model 5 is conducted strictly on the Propensity Score‐Matched (PSM) cohort to minimize baseline confounding.

The analysis of the LAR and its association with the risk of delirium in HF patients was elucidated through various multivariate logistic regression models, as presented in Table 3 and illustrated in Figure 1. In the unadjusted model, the OR for delirium in patients with LAR > −0.38 was 2.062 (95% CI: 1.526–2.785, *p* < 0.001), indicating a substantial increase in delirium risk when compared to the reference group (LAR ≤ −0.84). This trend continued in Model 1, which yielded an OR of 1.525 (95% CI: 1.114–2.088, *p* = 0.008) for those with LAR values between −0.84 and −0.38, and 2.130 (95% CI: 1.570–2.889, *p* < 0.001) for the highest LAR group.

**Table 3:** Logistic regression model analysis showed the relationship between LAR and delirium among different groups.

	LAR		LAR ( ≤ −0.84)	LAR (−0.84−0.38)		LAR ( > −0.38)		*P* for trend
Model	OR (95%CI)	*p*		OR (95%CI)	*p*	OR (95%CI)−2	*p*	
Unadjusted model	1.511 (1.266–1.802)	< 0.001	Reference	1.488 (1.090–2.032)	0.012	2.062 (1.526–2.785)	< 0.001	< 0.001
Model 1	1.539 (1.287–1.841)	< 0.001	Reference	1.525 (1.114–2.088)	0.008	2.130 (1.570–2.889)	< 0.001	< 0.001
Model 2	1.581 (1.320–1.895)	< 0.001	Reference	1.543 (1.126–2.116)	0.007	2.202 (1.620–2.994)	< 0.001	< 0.001
Model 3	1.518 (1.249–1.845)	< 0.001	Reference	1.518 (1.103–2.088)	0.010	2.080 (1.504–2.876)	< 0.001	< 0.001
Model 4	1.596 (1.306–1.951)	< 0.001	Reference	1.539 (1.115–2.123)	0.009	2.212 (1.590–3.078)	< 0.001	< 0.001
Model 5	1.305 (1.039–1.639)	0.022	Reference	1.429 (1.014–2.014)	0.042	1.655 (1.151–2.381)	0.007	0.007

Abbreviations: CI, Confidence Interval; OR, Odds Ratio.

Models 2 through 4 consistently demonstrated statistically significant odds ratios for increased LAR levels, reinforcing the linear relationship between elevated LAR and delirium risk. Specifically, Model 2 showed an OR of 1.581 (95% CI: 1.320–1.895, *p* < 0.001) for the middle LAR group and 2.202 (95% CI: 1.620–2.994, *p* < 0.001) for those with LAR > −0.38. Model 3 yielded an OR of 1.518 (95% CI: 1.249–1.845, *p* < 0.001) for LAR (−0.84 to −0.38) and 2.080 (95% CI: 1.504–2.876, *p* < 0.001) for higher LAR values, while Model 4 reported an OR of 1.596 (95% CI: 1.306–1.951, *p* < 0.001) for the middle cohort and 2.212 (95% CI: 1.590–3.078, *p* < 0.001) for the highest category.

Interestingly, in Model 5, the odds ratio for LAR ≤ −0.84 was reduced to 1.305 (95% CI: 1.039–1.639, *p* = 0.022); however, the middle and higher LAR categories still yielded significant odds, with ORs of 1.429 (95% CI: 1.014–2.014, *p* = 0.042) and 1.655 (95% CI: 1.151–2.381, *p* = 0.007), respectively. The overall trend across all models, with a p‐value for trend consistently below 0.001, demonstrates a clear and robust correlation between elevated LAR levels and an increased risk of delirium.

### 3.4 Factors Influencing Delirium Risk in Heart Failure Patients

**Figure 2:**
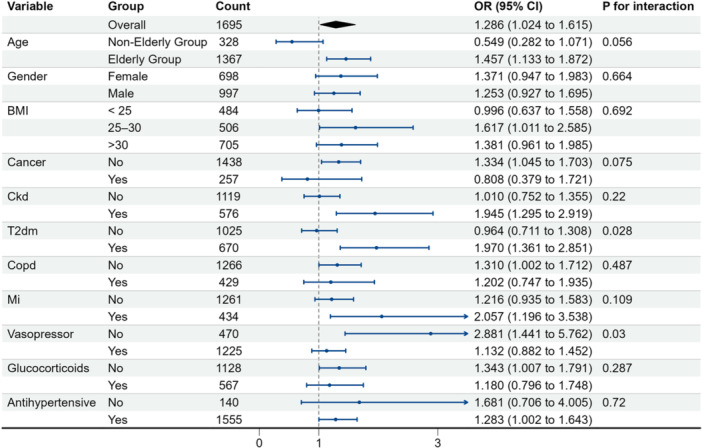
Associations between demographic and clinical characteristics and delirium risk (forest plot). Odds ratios (ORs) and 95% confidence intervals are shown for age, sex, BMI, cancer, CKD, T2DM, COPD, prior MI, and treatment‐related variables (vasopressors, glucocorticoids, antihypertensives).

The subgroup analysis presented in Figure 2 details the association between LAR and delirium risk across various demographic and clinical stratifications. In the overall cohort, an elevated LAR was significantly associated with an increased risk of delirium (OR: 1.286, 95% CI: 1.024–1.615).

When stratified by age, the association remained significant in the elderly group (OR: 1.457, 95% CI: 1.133–1.872), whereas it was not significant in the non‐elderly group (OR: 0.549, 95% CI: 0.282–1.071). Regarding BMI, the risk was notably elevated in patients with a BMI between 25 and 30 (OR: 1.617, 95% CI: 1.011–2.585).

The presence of specific comorbidities also influenced the risk profile. The association between higher LAR and delirium was particularly pronounced in patients with chronic kidney disease (CKD) (OR: 1.945, 95% CI: 1.295–2.919), type 2 diabetes mellitus (T2DM) (OR: 1.970, 95% CI: 1.361–2.851), and a history of myocardial infarction (MI) (OR: 2.057, 95% CI: 1.196–3.538). In terms of treatment approaches, patients not receiving vasopressors showed a robust association between LAR and delirium (OR: 2.881, 95% CI: 1.441–5.762).

Notably, the P for interaction analyses indicated that the predictive value of LAR for delirium was generally consistent across most subgroups, as most interaction *P*‐values were > 0.05. Significant interactions were observed only for T2DM (P for interaction = 0.028) and vasopressor use (P for interaction = 0.030), suggesting that the impact of LAR on delirium risk might be modified by diabetic status and hemodynamic support requirements.

### 3.5 Model Explanation

**Figure 3:**
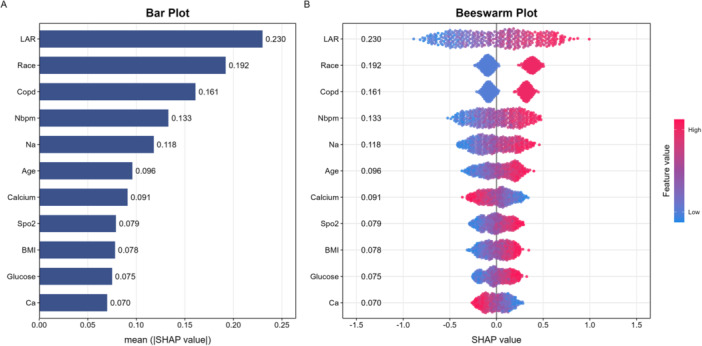
Feature importance of the delirium risk prediction model (SHAP). (A) Bar plot of mean absolute SHAP values; (B) SHAP beeswarm plot. LAR shows the highest contribution, followed by race, COPD, Nbpm, serum sodium (Na), age, etc.

The analysis presented in Figure 3A,B explores the importance of various clinical features in predicting delirium risk in heart failure patients, utilizing SHAP (SHapley Additive exPlanations) values. In the Bar Plot, the LAR stands out with the highest mean SHAP value of 0.230, indicating it as the most significant predictor of delirium risk among the assessed variables. Following LAR, race shows a substantial mean SHAP value of 0.192, suggesting its notable role in influencing delirium risk. COPD ranks next with a mean SHAP value of 0.161, further emphasizing its contribution. Other significant features include the number of beats per minute (Nbpm) at 0.133, sodium (Na) at 0.118, and age at 0.096. While calcium, oxygen saturation (Spo2), body mass index (BMI), glucose, and calcium (Ca) exhibit lower mean SHAP values ranging from 0.070 to 0.091, they still contribute to the model, albeit to a lesser extent. The Beeswarm Plot provides a visual distribution of the SHAP values for each feature, where the horizontal position indicates each feature's contribution to the prediction. LAR not only has the highest average value but also shows a wide range of SHAP values, implying variability in its influence across different patients. This trend is similarly observed for race and COPD, indicating that certain demographic and clinical factors can vary significantly in impact based on individual patient circumstances. Overall, both plots emphasize the critical role of LAR in assessing delirium risk in heart failure patients, while also acknowledging the importance of other factors such as race and COPD. The findings encourage further investigation into the interplay of these features and their potential implications for tailored management strategies in vulnerable populations.

It is important to explicitly note that while SHAP values provide robust insights into feature importance and predictive contribution within the machine learning model, they do not establish causality. The high SHAP value of LAR indicates its strong predictive power for delirium risk, but further mechanistic studies are required to confirm causative links.

### 3.6 Mediation Analysis of LAR and Delirium Risk in Heart Failure Patients

The mediation analysis of the LAR provides insightful revelations about the interplay between clinical mediators and the risk of delirium in heart failure patients (Table 4). The results indicate that higher LAR levels are significantly linked to reduced albumin levels and elevated white blood cell counts, suggesting that both nutritional deficits and inflammatory processes may play essential roles in mediating delirium risk.

**Table 4:** Mediation analysis of LAR, mediating factors, and delirium risk.

Mediator	LAR relationship	Delirium risk relationship	Adjusted odds ratio (OR)	95% Confidence interval (CI)	*p*
Albumin	down	down	1.305	(1.039–1.639)	0.022
White blood cell count	up	up	1.945	(1.295–2.919)	< 0.001
Creatinine	up	up	2.080	(1.504–2.876)	< 0.001
C‐Reactive protein	up	up	1.752	(1.310–2.345)	< 0.001
Blood Uuea Nitrogen	up	up	1.834	(1.256–2.678)	< 0.001
Age	up	up	1.457	(1.133–1.872)	0.056
BMI	—	—	1.381	(0.961–1.985)	0.692
Vasopressor use	up	up	2.881	(1.441–5.762)	0.03

Abbreviations: CI, Confidence Interval; OR, Odds Ratio.

Notably, the analysis reveals that for each unit increase in white blood cell count, the risk of developing delirium increases by 1.945 times (OR: 1.945, 95% CI: 1.295–2.919, *p* < 0.001), highlighting the potential importance of inflammation in this patient population. Similarly, creatinine levels exhibit a strong association with delirium risk, showing an odds ratio of 2.080 (95% CI: 1.504–2.876, *p* < 0.001). This suggests that renal function may influence cognitive outcomes, possibly due to the accumulation of toxic metabolites that affect brain function.

Age emerges as a significant factor, with older patients facing a 1.457‐fold increase in delirium risk (OR: 1.457, 95% CI: 1.133–1.872, *p* = 0.056). This aligns with existing literature indicating that advanced age is a critical vulnerability factor in cognitive impairment among heart failure patients. Although body mass index (BMI) did not show a significant relationship with delirium risk, the requirement for vasopressor support was strongly associated with a 2.88‐fold increased risk of delirium (OR: 2.881, 95% CI: 1.441–5.762, *p* = 0.03). This underscores the complexities of managing critically ill patients, where hemodynamic instability may contribute to cognitive decline.

Overall, this analysis emphasizes the vital mediating roles that nutritional status and inflammatory markers can play in the relationship between LAR and delirium risk. By understanding these interactions, clinicians might better identify at‐risk populations and develop targeted interventions to improve cognitive outcomes in heart failure patients. These findings not only contribute to the existing body of literature but also pave the way for future research aimed at mitigating delirium through addressing the identified mediating factors.

## 4 Discussion

The findings of this study provide important insights into the association between the LAR and the risk of delirium in patients with acute HF in an ICU setting. Given the high incidence of delirium observed in critically ill patients, understanding potential biomarkers like LAR can help identify individuals at increased risk, enabling timely intervention.

### 4.1 Key Findings

Our study demonstrated a significant correlation between elevated LAR values and the incidence of delirium among critically ill heart failure patients. This relationship aligns with existing literature that seeks to link metabolic derangements and nutritional deficits with cognitive dysfunction in hospitalized patients. Higher levels of lactate reflect tissue hypoperfusion, often indicative of underlying metabolic stress, while lower levels of serum albumin typically signal poor nutritional status. Both conditions are prevalent in patients with acute heart failure, particularly those experiencing acute decompensation.

The analysis also highlighted the role of comorbidities as mediating factors influencing delirium risk. Age, BMI, and specific chronic illness histories, such as COPD and T2DM, were associated with varying degrees of delirium risk. These findings suggest that clinicians should consider both metabolic derangements indicated by LAR and broader patient health profiles when assessing delirium risk.

### 4.2 Mechanisms of Delirium Development

It is crucial to emphasize that LAR should not be interpreted as a disease‐specific or delirium‐specific biomarker. Rather, it serves as a reliable proxy for global physiological stress and systemic vulnerability in a highly heterogeneous acute heart failure population. The robust association between elevated LAR and delirium likely reflects shared upstream pathological pathways—namely, systemic hypoperfusion, uncontrolled inflammation, and profound metabolic stress—which collectively predispose these critically ill patients to acute cognitive dysfunction [24]. Metabolic Derangement: Elevated lactate levels typically reflect tissue hypoperfusion and cellular hypoxia driven by inadequate oxygen delivery during cardiac dysfunction [25, 26, 27]. This hypoxic state can trigger neurochemical imbalances and neuronal excitotoxicity, which subsequently culminate in cognitive impairment and delirium [28, 29]. The brain is particularly sensitive to changes in metabolic status; alterations in blood flow or oxygenation can lead to impaired neurotransmitter functioning, contributing to the acute confusion characteristic of delirium [30].

Nutritional Deficits: Low serum albumin reflects poor nutritional status, which is crucial for maintaining brain homeostasis [31]. Albumin plays a role in transporting various substances, including hormones and medications, influencing their availability and efficacy [32, 33]. Low albumin levels may also suggest a state of increased systemic inflammation, further contributing to brain dysfunction. In malnourished states, the brain may be deprived of essential nutrients that support cognitive function, exacerbating the risk of delirium [34].

Inflammation: The interplay between elevated lactate and low albumin is often indicative of a systemic inflammatory response, commonly encountered in acute heart failure patients [35, 36]. Inflammatory cytokines can affect neurotransmitter systems and promote neuroinflammation, both of which are associated with delirium [37]. Elevated levels of inflammatory markers such as C‐reactive protein (CRP) have been shown to correlate with delirium severity, emphasizing the role of systemic inflammation in cognitive impairment [38, 39].

Neuroendocrine Response: Stress responses triggered by acute illness can activate the hypothalamic‐pituitary‐adrenal (HPA) axis, leading to elevated cortisol levels [40]. While cortisol is essential for the stress response, excessively high levels may lead to neuronal damage and impair synaptic plasticity, contributing to cognitive disturbances seen in delirium [41, 42].

### 4.3 Delirium Within the Continuum of Cognitive Impairment

Furthermore, delirium in acute heart failure should not be viewed as an isolated clinical event, but rather as an acute manifestation of underlying cerebral vulnerability within a broader continuum of cognitive impairment. Recent literature has extensively highlighted the bidirectional relationship between heart failure and cognitive dysfunction across the disease spectrum [6]. As summarized in a recent narrative review, chronic heart failure structurally and functionally alters the brain, lowering the threshold for acute cognitive failure when systemic stressors occur. In this context, an elevated LAR highlights the peak of this physiological stress, triggering delirium in a brain already sensitized by chronic cardiovascular compromise.

### 4.4 Implications for Clinical Practice

The implications of these findings are far‐reaching. By integrating LAR into routine clinical assessments, healthcare providers might better stratify patients' risk profiles for delirium. However, it is imperative to emphasize that biomarker‐based risk stratification tools like LAR are designed to complement, rather than replace, comprehensive clinical reasoning and clinician‐led assessments. Implementing early intervention measures for those identified as high‐risk could potentially reduce the incidence of delirium and improve overall patient outcomes. For instance, targeted nutritional support and hemodynamic optimization may help mitigate the metabolic and nutritional deficits that contribute to delirium onset.

Furthermore, our study underscores the importance of a multidisciplinary approach in managing critically ill patients. Involving dietitians, pharmacists, and critical care specialists would foster comprehensive treatment plans that address not only the immediate cardiac issues but also the metabolic and nutritional needs of patients, ultimately enhancing cognitive outcomes.

### 4.5 Limitations

Several limitations should be noted. First, the retrospective design inherently limits causal inference; therefore, the observed correlation between LAR and delirium risk does not establish a direct causative relationship. Additionally, the analysis relied on data from a single database, which, while large and diverse, may not fully capture the variability present in other healthcare settings. Another notable limitation is our reliance on ICD‐9 and ICD‐10 billing codes for the diagnoses of acute heart failure and delirium, without direct access to confirmatory clinical data such as specific natriuretic peptide levels or standardized delirium assessment tools (e.g., CAM‐ICU). Consequently, potential coding errors or misclassification bias cannot be completely ruled out, which may affect the generalizability of our findings. Furthermore, while we excluded patients with a documented history of dementia, our analysis did not account for other baseline neurological vulnerabilities, such as mild cognitive impairment (MCI) or a history of transient ischemic attack (TIA) and cerebrovascular accident (CVA). These unmeasured confounders are established risk factors for delirium and could potentially influence the observed association between LAR and delirium risk.

### 4.6 Future Research Directions

Future studies should aim to validate our findings in diverse cohorts outside of the MIMIC‐IV database. Prospective studies are necessary to establish causality between LAR and delirium and to understand the underlying mechanisms driving this relationship. Research should also focus on the development and testing of tailored intervention strategies that target both metabolic derangements and cognitive outcomes in acute heart failure patients.

Moreover, greater emphasis should be placed on exploring potential interactions between biochemical markers, systemic inflammation, and psychological factors, such as anxiety and depression, which may also influence delirium development. Understanding these relationships may provide even deeper insights into the complex interplay between physiological stress and cognitive function in critically ill patients.

## 5 Conclusion

In conclusion, this study elucidates the significant role of the lactate/albumin ratio as a potential biomarker for delirium risk in acute heart failure patients. By highlighting the interplay between metabolic status, nutritional health, and delirium development, our findings underscore the necessity of a multifaceted approach in managing critically ill patients. Clinicians can better identify those at risk and implement timely and effective interventions to reduce the burden of delirium in this vulnerable population, ultimately improving patient outcomes in the ICU setting.

## Author Contributions

All authors contributed to the article; Wenjuan Yan: Formal analysis, Conceptualization, Software. Yajuan Wang Wrote the manuscript. Qiulan Wen and Pengwei Shi: Supervision and revising the manuscript.

## Funding

The authors have nothing to report.

## Ethics Statement

The study was conducted in accordance with the Declaration of Helsinki (2013 revision). Access to and use of the de‐identified MIMIC‐IV (v3.1) database were approved by the Institutional Review Boards of the Massachusetts Institute of Technology and Beth Israel Deaconess Medical Center, with a waiver of informed consent. As this secondary analysis used only de‐identified, publicly available data, no additional local institutional review board approval was required.

## Conflicts of Interest

The authors declare that the research was conducted in the absence of any commercial or financial relationships that could be construed as a potential conflict of interest.

## Data Availability

The data used in the present study are all publicly available at https://physionet.org/content/mimiciv/3.1/.
